# *In vitro* evaluation of antifungal combinations against neurotropic dematiaceous fungi associated with primary cerebral phaeohyphomycosis

**DOI:** 10.1128/spectrum.00781-24

**Published:** 2024-06-26

**Authors:** Arghadip Samaddar, Jenevi Margaret Mendonsa, Sukanya Sudhaharan, Nagarathna S, Anupma Jyoti Kindo, Anjali Shetty, Umabala Pamidimukkala

**Affiliations:** 1 Department of Neuromicrobiology, National Institute of Mental Health and Neuro Sciences, Bengaluru, Karnataka, India; 2 Department of Microbiology, ICMR-Advanced Mycology Diagnostic and Research Centre, Nizam’s Institute of Medical Sciences, Hyderabad, Telangana, India; 3 Department of Microbiology, Sri Ramachandra Institute of Higher Education and Research, Chennai, Tamil Nadu, India; 4 Department of Microbiology, ICMR-Advanced Mycology Diagnostic and Research Centre, P.D. Hinduja Hospital and Medical Research Centre, Mumbai, Maharashtra, India; Jawaharlal Nehru Centre for Advanced Scientific Research, Bangalore, Karnataka, India

**Keywords:** cerebral phaeohyphomycosis, dematiaceous fungi, antifungal susceptibility testing, checkerboard assay, *Cladophialophora bantiana*, *Fonsecaea monophora*, *Cladosporium cladosporioides*

## Abstract

Primary cerebral phaeohyphomycosis is a life-threatening disease caused by neurotropic dematiaceous fungi. At present, there are no consensus guidelines regarding optimal antifungal therapy in such cases. Generally, a combination of antifungal agents is recommended for treatment. However, the activities of antifungal combinations against these fungi have not been investigated. In this study, we evaluated the *in vitro* activities of 13 double and five triple antifungal combinations against clinical isolates of *Cladophialophora bantiana* (*n* = 7), *Fonsecaea monophora* (*n* = 2), and *Cladosporium cladosporioides* (*n* = 1), using a simplified checkerboard procedure. The minimum inhibitory concentrations (MICs) of nine antifungal drugs were determined by the broth microdilution method, and the interaction between antifungal agents in each combination was assessed by the fractional inhibitory concentration index. Excellent activity was observed for posaconazole and itraconazole. Flucytosine had potent activity against *C. bantiana* but was ineffective against *F. monophora*, and *C. cladosporioides*. The echinocandins demonstrated high MICs for all the isolates. Synergistic interactions were observed for all the double combinations, except when itraconazole was combined with either amphotericin B or flucytosine. The combination of amphotericin B with caspofungin showed synergistic interactions against 40% of the isolates. Antagonism was observed with isavuconazole-flucytosine combination against two *C. bantiana* isolates. The triple combinations of caspofungin and flucytosine with amphotericin B or posaconazole were synergistic against one isolate of *F. monophora*. For *C. cladosporioides*, synergy was observed for the triple combination of amphotericin B with caspofungin and flucytosine. Our results indicate that combination of caspofungin with amphotericin B or a triazole, with or without 5-flucytosine has great potential against neurotropic dematiaceous fungi.

**IMPORTANCE:**

This research uses a modified version of the checkerboard assay to standardize the *in vitro* testing of double and triple combinations of antifungal agents against neurotropic dematiaceous fungi. Antifungal combination therapy is associated with improved outcomes in cerebral phaeohyphomycosis. In this study, we demonstrate that posaconazole is the single most active antifungal drug against this group of fungi. The double combination of amphotericin B with caspofungin or a trizole, and the triple combinations of caspofungin and flucytosine with amphotericin B or posaconazole might hold promise in the treatment of cerebral phaeohyphomycosis. Our findings will guide in developing optimal therapeutic strategies for these refractory infections.

## INTRODUCTION

Phaeohyphomycosis refers to infections caused by fungi that contain melanin in their cell walls. These fungi are ubiquitous saprophytes, inhabiting soil, and decaying plant material (1). They can cause a wide spectrum of manifestations, ranging from subcutaneous lesions to fatal infections, such as brain abscess, pneumonia, and disseminated disease (1, 2). Primary cerebral phaeohyphomycosis (CPHM) refers to infections of the brain parenchyma caused by dematiaceous fungi. Unlike other filamentous fungal infections that are associated with severe immunocompromise, the disease predominantly affects healthy individuals, with no underlying risk factors (3, 4). CPHM has a worldwide distribution, with a considerable number of cases being reported from Asian countries, especially from India (4). There has been an exponential increase in the number of cases during the last two decades. Most of the causative agents belong to a single order, *Chaetothyriales*, with *Cladophailophora bantiana* accounting for a majority of the cases. Others include *Rhinocladiella mackenziei*, *Verruconis gallopava*, *Fonsecaea monophora*, *Exophiala dermatitidis*, *Nodulisporium* spp., and *Rhinocladiella atrovirens* (2, 3). Though fungi are uncommon causes of central nervous system (CNS) infections, agents of CPHM have a particular predilection for the brain. However, the mechanisms for neurotropism remain unclear.

The clinical management of CPHM is challenging due to poor response to the existing antifungal regimens. In the absence of standard therapy, the management considerations are largely based on data from retrospective case reviews and anecdotal case reports. The European Confederation of Medical Mycology and European Society of Clinical Microbiology and Infectious Diseases joint guidelines recommend complete excision of the abscess, together with antifungal mono or combination therapy (5). However, despite combined treatment with surgery and antifungal drugs, the prognosis remains poor with mortality rates approaching 70%, regardless of the immune status of the individual (3). The treatment of invasive fungal infections primarily relies upon four major classes of antifungal drugs: polyenes, azoles, echinocandins, and the nucleoside analog 5-flycytosine. Among these agents, 5-flucytosine and newer triazoles, such as voriconazole, posaconazole, and itraconazole demonstrate potent *in vitro* activity against dematiaceous fungi and achieve high concentrations in the brain parenchyma (1, 4). Though clinical improvement has been observed with these antifungals in some series, the *in vitro* susceptibility patterns of this group of fungi are highly variable and there are no defined interpretive breakpoints to guide optimal antifungal therapy (4, 5). The combination of amphotericin B and 5-flucytosine, commonly used for the treatment of CNS fungal infections, failed to improve survival in CPHM, as observed in several studies (3, 4). The triple combination of a triazole with 5-flucytosine and echinocandin is associated with improved survival and is recommended as the first-line therapy when surgery is not possible (5). However, the *in vitro* effectiveness of such combinations has not been elucidated. Therefore, the present study aimed to investigate the *in vitro* activities of nine major antifungal drugs (amphotericin B, 5-flucytosine, voriconazole, posaconazole, itraconazole, isavuconazole, caspofungin, micafungin, and anidulafungin) and their combinations against neurotropic dematiaceous fungi associated with primary CPHM. Our findings will guide in developing appropriate therapeutic strategies and thus, improve the outcomes in such cases.

## MATERIALS AND METHODS

### Fungal isolates

A collection of 10 clinical isolates of neurotropic dematiaceous fungi, consisting of 7 strains of *C. bantiana*, 2 strains of *F. monophora*, and 1 strain of *C. cladosporioides*, obtained from human CPHM cases were included in the study. The isolates were collected from various centers across India (Table 1) and were handled in a Biosafety Level 2 containment facility with appropriate personal protective equipment, as recommended by the Centers for Disease Control and Prevention, USA (6). They were grown on potato dextrose agar (supplemented with 0.02% chloramphenicol) for 7 days at 25°C. Colony characteristics and microscopic morphologies were recorded to ensure purity and viability. The identification at the species level was confirmed by sequencing the internal transcribed spacer region of ribosomal DNA, as described previously (7). The sequences were submitted to the GenBank database and published in the NCBI database on 14 March 2024, with the accession numbers from PP462145 to PP462154 (Table 1). For the estimation of cardinal growth temperatures, the isolates were grown for 14 days at temperatures ranging from 25°C to 45°C at intervals of 5°C, as well as at 32°C and 37°C.

**TABLE 1:** Origin and source of fungal isolates included in the study

Isolate no	Species	Origin	Source	Site of lesion	GenBank accession nos.^*a*^
CPRNIMS-01	*C. bantiana*	NIMS, Hyderabad^*b*^	Brain abscess	Frontal	PP462145
CPRNIMS-02	*C. bantiana*	NIMS, Hyderabad	Brain abscess	Temporal	PP462146
CPPNIMS-01	*C. bantiana*	NIMS, Hyderabad	Brain abscess	Fronto-parietal	PP462147
CPRSRMC-01	*C. bantiana*	SRIHER, Chennai^*c*^	Brain abscess	Frontal, parietal	PP462148
CPRHIND-01	*C. bantiana*	P.D. Hinduja Hospital, Mumbai	Brain abscess	Frontal	PP462149
CPRHIND-02	*C. bantiana*	P.D. Hinduja Hospital, Mumbai	Brain abscess	Fronto-parietal	PP462150
CPPAIIMS	*C. bantiana*	AIIMS, Jodhpur^*d*^	Brain abscess	Basal ganglia	PP462151
CPRSRMC-02	*F. monophora*	SRIHER, Chennai	Brain abscess	Fronto-parietal	PP462152
CPPNIMHANS-01	*C. cladosporioides*	NIMHANS, Bengaluru^*e*^	Brain abscess	Brain stem	PP462153
CPRNIMHANS-02	*F. monophora*	NIMHANS, Bengaluru	Brain abscess	Frontal	PP462154

^*a*^ Reference to GenBank repository with accession numbers for the submitted sequences.

^*b*^ NIMS, Nizam’s Institute of Medical Sciences.

^*c*^ SRIHER, Sri Ramachandra Institute of Higher Education and Research.

^*d*^ AIIMS, All India Institute of Medical Sciences.

^*e*^ NIMHANS, National Institute of Mental Health and Neuro Sciences.

### Antifungal susceptibility testing

Antifungal susceptibility testing was performed using the broth microdilution method, according to the Clinical and Laboratory Standards Institute M38A2 document (8). Sterile polystyrene round bottom 96-well microtiter plates were used. RPMI 1640 with L-glutamine but without sodium bicarbonate and buffered to pH 7.0 with 0.165 moles/L morpholinepropanesulfonic acid (HiMedia Labs Pvt. Ltd., Mumbai) was used as the test medium. Stock spore suspension was prepared by washing the surface of the slant with 1 mL of 0.85% sterile saline and 10 uL of tween 20. The colonies were gently probed with the tip of a transfer pipette and the suspension was aspirated in a sterile test tube. After allowing heavy particles to settle for 3 to 5 minutes, the upper homogeneous suspension was transferred to a sterile tube and mixed for 15 seconds. The density of the conidial suspension was adjusted spectrophotometrically at 530 nm to an optical density ranging from 0.15 to 0.17. The suspension was diluted 1:50 (for double combinations) or 1:25 (for triple combinations) with RPMI 1640 medium in a sterile reagent reservoir to achieve a 2× or 4× concentration of the required density (0.5–3.1 × 10^4^ CFU/mL), respectively. The antifungal agents included amphotericin B (AmB), voriconazole (VRC), posaconazole (PSC), isavuconazole (ISA), itraconazole (ITR), 5-flucytosine (5-FC), caspofungin (CFG), micafungin (MFG), and anidulafungin (AFG) (Sigma-Aldrich, Germany). Stock solutions were prepared by dissolving the drugs in 100% dimethyl sulfoxide (DMSO). For minimum inhibitory concentration (MIC) distribution, the range of antifungal concentrations tested was 0.015 to 16 µg/mL for AmB, VRC, PSC, ISA, and ITR, 0.06 to 64 µg/mL for 5-FC, and 0.008 to 8 µg/mL for CFG, MFG, and AFG. Standard strains of *Paecilomyces variotii* (ATCC 22319), *Candida parapsilosis* (ATCC 22019), and *Candida krusei* (ATCC 6258) were used as quality controls in all the experiments.

### Two-dimensional checkerboard assay for double antifungal combinations

**Fig 1:**
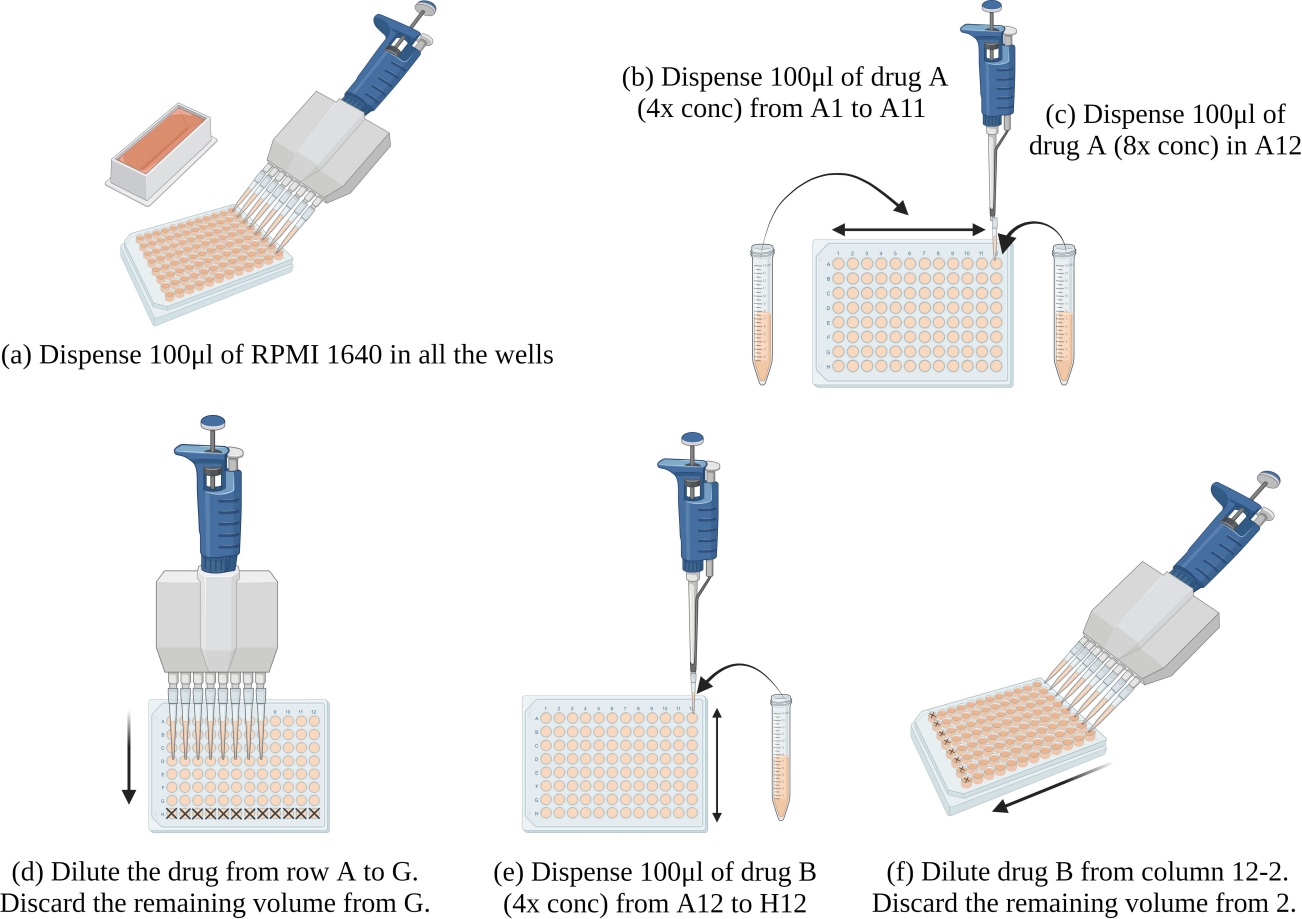
Schematic set-up of two-dimensional checkerboard assay for double antifungal combinations. The fractional inhibitory concentration index (FICI) values were determined for each of the 13 combinations: AmB/5-FC, AmB/VRC, AmB/PSC, AmB/ISA, AmB/CFG, AmB/ITR, PSC/5-FC, ISA/5-FC, ISA/CFG, VRC/CFG, VRC/MFG, ITR/AFG, and ITR/5-FC. Steps (a) to (f) demonstrate serial dilutions of two drugs in the checkerboard.

Stock solutions of antifungal drugs were prepared in DMSO at 100 times the highest concentration tested. A simplified checkerboard method for testing antifungal combinations was developed, as a modification of the procedure mentioned in Clinical Microbiology Procedures Handbook, 3rd edition (9). The protocol was standardized by testing the reference strains of *P. variotii* ATCC 22319, *C. parapsilosis* ATCC 22019, and *C. krusei* ATCC 6258 in triplicates. A two-dimensional checkerboard with a twofold dilution of each drug was set up [Tables S1 to S8 (supplemental material can be found at https://doi.org/10.6084/m9.figshare.26015212.v2)]. First, 100 µL of RPMI 1640 was dispensed in 96-well microtiter plates. Then, stock solutions of the first drug (Drug A) that were fourfold (4×) and eightfold (8×) more concentrated than the highest concentration tested were prepared in RPMI 1640. Thereafter, 100 uL of 4× solution of Drug A was dispensed in row A, from A1 to A11 of the microtiter plate, and 100 uL of 8× solution was dispensed in the well A12 (Table S1). The drug was then serially diluted from row A to G, using a multichannel pipette set at 100 uL dispensing volume, discarding the remaining volume from row G (Table S2). A stock solution of the second drug (Drug B) that was 4× more concentrated than the highest concentration tested was prepared in RPMI 1640. Then, 100 uL of the above stock solution was dispensed into the 12th column of the microtiter plate from A12 to H12, and serially diluted from column 12 to 2, using an 8-channel pipette set at 100 uL dispensing volume, discarding the remaining volume from column 2 (Table S3). The same procedure was followed for all the drug combinations. The well H1 served as the drug-free growth control. Column 1, i.e., wells A1 to G1 represented the serial dilutions of Drug A alone, with A1 having 2× more concentration than the highest concentration of Drug A tested. Wells H12 to H2 represented serial dilutions of Drug B alone, with H12 having 2× more concentration than the highest concentration of Drug B tested (Fig. 1). A total of 13 double antifungal combinations were tested against *C. bantiana*, *F. monophora*, and *C. cladosporioides*. For the double combinations of AmB with either VRC, PSC, ISA, ITR, 5-FC, or CFG, the final concentrations of the antifungal agents were 0.25 to 16 µg/mL for AmB (i.e., 7 twofold dilutions), 0.016 to 16 µg/mL for VRC, PSC, ISA, and ITR (i.e., 11 twofold dilutions) (Table S4), 0.06 to 64 µg/mL for 5-FC (i.e., 11 twofold dilutions) (Table S5), and 0.008 to 8 µg/mL for CFG (i.e., 11 twofold dilutions) (Table S6). For the double combination of PSC or ITR with 5-FC, the final concentrations of the antifungal agents were 0.25 to 16 µg/mL for PSC and ITR (i.e., 7 twofold dilutions), and 0.06 to 64 µg/mL for 5-flucytosine (i.e., 11 twofold dilutions) (Table S7). For the double combinations of VRC with either CFG or MFG, the final concentrations of the antifungal agents were 0.25 to 16 µg/mL for VRC (i.e., 7 twofold dilutions), and 0.008 to 8 µg/mL for CFG and MFG (i.e., 11 twofold dilutions) (Table S8). For the double combinations of ISA with either CFG or 5-FC, the final concentrations of the antifungal agents were 0.25 to 16 µg/mL for ISA (i.e., 7 twofold dilutions), 0.008 to 8 µg/mL for caspofungin (i.e., 11 twofold dilutions), and 0.06 to 64 µg/mL for 5-flucytosine (i.e., 11 twofold dilutions) (Tables S7 and S8). For the double combination of ITR with AFG, the final concentrations of the antifungal agents were 0.25 to 16 µg/mL for ITR (i.e., 7 twofold dilutions), and 0.008 to 8 µg/mL for AFG (i.e., 11 twofold dilutions) (Table S8).

### Three-dimensional checkerboard assay for triple antifungal combinations

**Fig 2:**
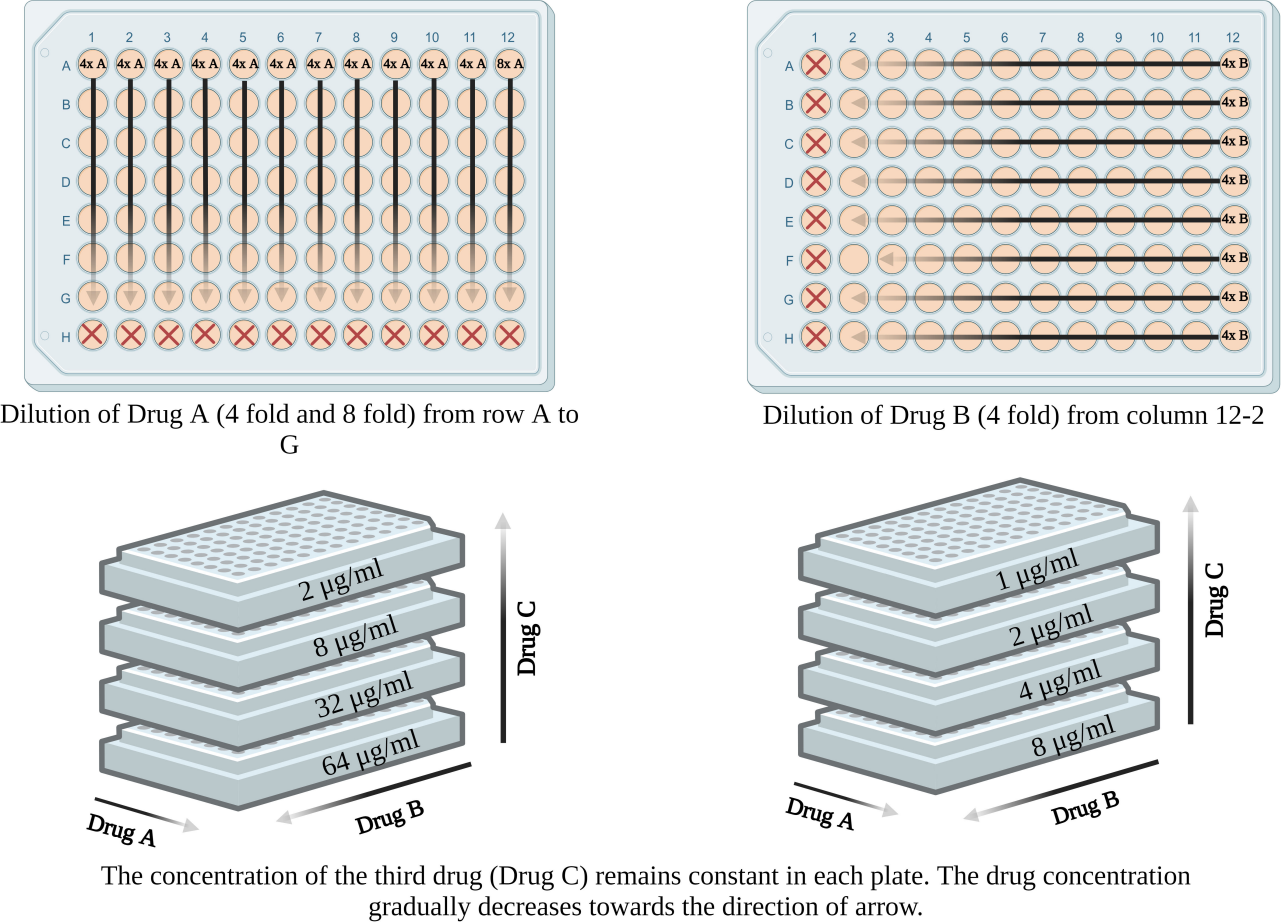
Schematic set-up of three-dimensional checkerboard assay for triple antifungal combinations. The concentration of each drug decreases toward the direction of the arrow. The FICI values were determined for each of the five combinations: AmB/CFG/5-FC, VRC/CFG/5-FC, PSC/CFG/5-FC, AmB/ITR/5-FC, and AmB/ITR/AFG.

The triple combinations, AmB with CFG and 5-FC, VRC with CFG and 5-FC, PSC with CFG and 5-FC, AmB with ITR and 5-FC, and AmB with ITR and AFG were tested against *C. bantiana*, *F. monophora*, and *C. cladosporioides* by a modified three-dimensional checkerboard method. A checkerboard with twofold dilutions of CFG and either AmB, VRC, or PSC was set up, as described for the double combinations. Then, 50 µL of 5-FC (4× more concentrated than the final concentration tested) was added at a single concentration per plate [Tables S9 to S20 (supplemental material can be found at https://doi.org/10.6084/m9.figshare.26015212.v2)]. Five plates were used for each isolate, including one plate without 5-FC. The final concentrations of the antifungal agents were 0.008 to 8 µg/mL for CFG, 0.25 to 16 µg/mL for AmB, VRC, and PSC, and 2 to 64 µg/mL for 5-FC (i.e., five concentrations were tested for 5-FC: 0, 2, 8, 32, and 64 µg/mL) (Tables S9 to S12). For the triple combinations of AmB with ITR and 5-FC, and AmB with ITR and AFG, a checkerboard with twofold dilutions of AmB and ITR was first set up. Then, 50 µL of either 5-FC or AFG (4× more concentrated than the final concentration tested) was added at a single concentration per plate. Five plates were used for each isolate for either combination, including one plate without 5-FC or AFG. The final concentrations of the antifungal agents were 0.25 to 16 µg/mL for AmB, 0.016 to 16 µg/mL for ITR, 2 to 64 µg/mL for 5-FC (i.e., five concentrations were tested for 5-FC: 0, 2, 8, 32, and 64 µg/mL) (Tables S13 to S16), and 1–8 μg/mL for AFG (i.e., five concentrations were tested for AFG: 0, 1, 2, 4, and 8 µg/mL) (Fig. 2; Tables S17 to S20).

### Plate inoculation and incubation

For the two-dimensional checkerboard, each well of the microtiter plates containing 100 µL of the double drug combination was inoculated with 100 µL of the inoculum suspension. For the three-dimensional checkerboard, each well of the microtiter plates containing 150 µL of the triple drug combination was inoculated with 50 µL of the inoculum suspension. The plates were covered with sterile lids and incubated at 37°C for 7 days for *C. bantiana* and *F. monophora*. For *C. cladosporioides*, the plates were incubated at 25°C for 7 days, given the optimal growth requirements of *Cladosporium* and related taxa.

### Interpretation of MIC and MEC

The microtiter plates were read visually and the growth in each well was compared to that in the growth control well. The experiments were performed in duplicate. The MICs of AmB, VRC, PSC, ITR, and ISA were determined by the lowest drug concentrations that prevented any discernible growth (100% inhibition), whereas for flucytosine, a 50% reduction in growth compared to the drug-free control was used. For echinocandins, the minimal effective concentration (MEC) was determined by the minimal concentration that resulted in small, rounded, compact hyphal forms in comparison to the hyphal growth seen in the growth control well. However, as the other antifungals were not responsible for the same effect, the MEC was not considered for the evaluation of antifungal interactions. Therefore, the MIC was used instead of the MEC for the evaluation of the combined effects of the drugs. The MIC endpoints were defined as the lowest drug concentration (tested alone or in combination) that prevented any visible growth. The MIC_50_ and MIC_90_ values were derived by noting the MIC data for each antifungal in ascending arrays and selecting the median and 90th quartile of the MIC distribution, respectively. The *in vitro* activities of antifungal combinations were evaluated by calculating the summation fractional inhibitory concentration (ΣFIC) index, based on the Loewe additivity formula. For double antifungal combinations, the FIC index was calculated as ΣFIC = FIC_A_ + FI_CB_. For triple combinations, the FIC index was expressed as ΣFIC = FIC_A_ + FI_CB_ + FIC_C_, where the FIC of an individual drug was calculated as the MIC of the drug in the combination divided by the MIC of the drug alone. For double combinations, the interaction was considered synergistic for fractional inhibitory concentration index (FICI) ≤ 0.5, partially synergistic between >0.5 and <1.0, additive at 1.0, indifferent between >1.0 and <4, and antagonistic >4. For triple combinations, the FICI was interpreted as synergism, ≤0.75; additive, >0.75–3; indifference, >3–4; and antagonism, >4. Additionally, response surface analyses and matrix synergy plots based on the Bliss Independence model were employed to visualize the drug interaction within each combination. The Combenefit software v.2.021 was used to perform Bliss analysis (10).

## RESULTS

### MIC distributions of antifungal drugs for *C. bantiana*, *F. monophora*, and *C. cladosporioides*

The MIC values of AmB, VRC, PSC, ITR, ISA, 5-FC, CFG, MFG, and AFG for 10 clinical isolates (7 *C*. *bantiana,* 2 *F*. *monophora*, and 1 *C*. *cladosporioides*) are summarized in Table 2. The modal MIC for AmB, 5-FC, ITR, and ISA against *C. bantiana* was 0.5 µg/mL, while that for VRC and PSC were 1 µg/mL and 0.015 µg/mL, respectively. The most potent antifungal activity against *C. bantiana* was observed for PSC (MIC_50_/MIC_90_, 0.015/0.015 µg/mL; range, 0.015 to 0.06), and this was followed by 5-FC (MIC_50_/MIC_90_, 0.5/0.5 µg/mL; range, 0.125 to 0.5). The MIC ranges, modal MIC, MIC_50_, and MIC_90_ for 7 *C*. *bantiana* isolates against the tested antifungals are summarized in Table 3. For *F. monophora*, the most potent antifungal activity was observed for PSC (MIC range, 0.015–0.06 μg/mL), followed by VRC (MIC range, 0.25–0.5 μg/mL), ISA (MIC range, 0.5–1 μg/mL), and ITR (1 µg/mL). AmB and 5-FC demonstrated high MICs against both the isolates (MIC ranges, 1–2 μg/mL and 4–16 μg/mL, respectively). The most potent antifungal activity against *C. cladosporioides* was observed for PSC (MIC, 0.06 µg/mL), followed by VRC (MIC, 0.5 µg/mL), while ISA, ITR, and AmB demonstrated moderate activity (MIC, 1 µg/mL). A high MIC was observed for 5-FC (64 µg/mL). The echinocandins demonstrated consistently high MICs across all the isolates, with a modal MIC of 8 µg/mL, and were virtually ineffective against these neurotropic dematiaceous fungi (Table 2).

**TABLE 2:** *In vitro* activities of nine antifungal drugs against *C. bantiana*, *F. monophora*, and *C. cladosporioides*

Isolate	Species	MIC (µg/mL)								
		AmB	VRC	PSC	ITR	ISA	5-FC	CFG^*a*^	MFG^*a*^	AFG^*a*^
CPRNIMS-01	*C. bantiana*	1	1	0.06	1	2	0.5	8	8	8
CPRNIMS-02	*C. bantiana*	0.25	2	0.015	1	1	0.25	8	8	8
CPPNIMS-01	*C. bantiana*	0.5	1	0.015	0.5	1	0.125	8	8	8
CPRSRMC-01	*C. bantiana*	0.5	0.5	0.015	0.5	0.5	0.5	8	8	8
CPRHIND-01	*C. bantiana*	0.5	0.5	0.015	0.5	0.5	0.5	4	4	4
CPRHIND-02	*C. bantiana*	1	1	0.015	0.5	0.5	0.5	4	4	4
CPPAIIMS	*C. bantiana*	2	2	0.015	1	0.5	0.5	8	4	8
CPRSRMC-02	*F. monophora*	2	0.25	0.06	1	0.5	16	8	4	8
CPPNIMHANS-01	*C. cladosporioides*	1	0.5	0.06	1	1	64	8	8	8
CPRNIMHANS-02	*F. monophora*	1	0.25	0.015	1	1	4	8	2	8

^*a*^ Data for caspofungin, micafungin, and anidulafungin represent MECs (μg/mL).

**TABLE 3:** Antifungal MIC distribution for seven clinical isolates of *C. bantiana^a^*

Drug	MIC (µg/mL)																
	0.008	0.015	0.03	0.06	0.125	0.25	0.5	1	2	4	8	16	32	64	Range	MIC_50_^*b*^	MIC_90_^*c*^
AmB		0	0	0	0	1	3^*d*^	2	1	0	0	0			0.25–2	0.5	1
5-FC				0	1	1	5	0	0	0	0	0	0	0	0.125–0.5	0.5	0.5
VRC		0	0	0	0	0	2	3	2	0	0	0			0.5–2	1	2
PSC		6	0	1	0	0	0	0	0	0	0	0			0.015–0.06	0.015	0.015
ITR		0	0	0	0	0	4	3	0	0	0	0			0.5–1	0.5	1
ISA		0	0	0	0	0	4	2	1	0	0	0			0.5–2	0.5	1
CFG^*e*^	0	0	0	0	0	0	0	0	0	2	5				4.0–8	8	8
MFG^*e*^	0	0	0	0	0	0	0	0	0	3	4				4.0–8	4	8
AFG^*e*^	0	0	0	0	0	0	0	0	0	2	5				4.0–8	8	8

^*a*^ Blank cells represent the concentrations that were not tested for the antifungal agent.

^*b*^ MIC_50,_ MIC at which 50% of isolates were inhibited.

^*c*^ MIC_90_, MIC at which 90% of isolates were inhibited.

^*d*^ Modal MICs are indicated with gray highlighted cells.

^*e*^ Data for caspofungin, micafungin, and anidulafungin represent MECs (μg/mL).

### *In vitro* activities of double antifungal combinations against *C. bantiana*, *F. monophora*, and *C. cladosporioides*

A total of 13 double antifungal combinations were evaluated for each isolate, which included AmB combined with 5-FC, VRC, PSC, ISA, ITR, or CFG; 5-FC combined with PSC, ISA, or ITR; ISA combined with CFG; VRC combined with CFG or MFG; and ITR combined with AFG (Table 4). For *C. bantiana*, synergistic interactions were observed for all the combinations, except when AmB was combined with ITR, and when 5-FC was combined with AmB, ISA, or ITR. The combination of AmB with CFG was synergistic in 3 of 7 *C*. *bantiana* isolates (synergy, 2/7 isolates; partial synergy, 1/7 isolate), and additive in 4/7 isolates. The combination of VRC with CFG led to synergistic interactions in 3/7 *C*. *bantiana* isolates (synergy, 1/7 isolate; partial synergy, 2/7 isolates), indifference in 3/7 isolates, and additive interaction in a single isolate. The combinations of AmB with VRC, ISA with CFG, VRC with MFG, and ITR with AFG showed synergistic interactions in 2/7 isolates, each (synergy, 1/7; partial synergy, 1/7 for each combination). Additive interactions were observed in 4/7 *C*. *bantiana* isolates for each of the combinations of VRC with MFG, and ITR with AFG, and in 2/7 isolates for AmB combined with ISA or ITR; and for PSC with 5-FC. The combination of ITR with 5-FC showed additive interaction against a single isolate. The combination of AmB with 5-FC showed indifferent interactions in all *C. bantiana* isolates. The least favorable interaction was observed for the combination of ISA with 5-FC, with 2/7 isolates showing antagonism (mean FICI, 5), and the remaining isolates displaying indifferent interactions (modal FICI, 3). For *F. monophora* (*n* = 2), the combinations of ISA with 5-FC and AmB with PSC showed synergy (FICI, 0.5), and partial synergy (FICI, 0.74) against a single isolate, respectively. The interactions of other antifungal combinations were either additive or indifferent for both the isolates (Table 4). For *C. cladosporioides* (*n* = 1), synergy was observed for AmB with 5-FC and partial synergy for AmB combined with PSC or CFG. The interactions of other antifungal combinations were either additive or indifferent, and antagonism was not observed. The MIC and FICI ranges for which the antifungal combinations showed synergistic interactions are summarized in Table 5.

**TABLE 4:** FICI values for 13 double antifungal combinations against *C. bantiana*, *F. monophora*, and *C. cladosporioides*

Isolate	Species		FICI values of antifungal combinations^*a*^											
		AmB + 5-FC	AmB + VRC	AmB + PSC	AmB + ISA	AmB + CFG	AmB + ITR	PSC + 5-FC	ISA + 5-FC	ISA + CFG	VRC + CFG	VRC + MFG	ITR + AFG	ITR + 5-FC
CPRNIMS-01	*C. bantiana*	2	1.5	**0.56**	**0.5**	**0.5**	1.5	3	1.5	**0.53**	**0.53**	**0.53**	**0.53**	1.5
CPRNIMS-02	*C. bantiana*	1.25	1.5	2	1	1	1.5	1.25	3	1.02	1.125	1.03	1	1
CPPNIMS-01	*C. bantiana*	1.5	1.125	1.5	1.5	1.02	1	1.5	3	1.125	**0.52**	1.03	1.02	1.5
CPRSRMC-01	*C. bantiana*	2.5	1.5	1.5	1.5	**0.5**	1	**0.52**	**5**	**0.5**	1.125	1.25	**0.5**	1.5
CPRHIND-01	*C. bantiana*	1.125	**0.53**	1.5	1.5	1	1.5	1.126	2	1.25	1.25	1.02	1.25	2
CPRHIND-02	*C. bantiana*	2.5	1.5	1.5	1.03	1	1.5	1.25	3	1.02	1	1	1	2
CPPAIIMS	*C. bantiana*	3	**0.38**	1.125	1.5	**0.56**	1.5	1.126	**5**	2.125	**0.5**	**0.5**	1.03	2
CPRSRMC-02	*F. monophora*	1.125	1	**0.74**	1.126	1.25	1	1	1.5	1	1	2	1	2
CPRNIMHANS-02	*F. monophora*	1.5	1.5	1.25	1.5	1.01	1.25	1.02	**0.5**	1.25	1	1.25	**0.53**	1.25
CPPNIMHANS-01	*C. cladosporioides*	**0.5**	1	**0.75**	1.5	**0.56**	1.5	1	1.01	1.02	1	1.03	1.02	1

^*a*^ FICI ≤ 0.5, synergy; FICI > 0.5 and <1.0, partial synergy; FICI = 1.0, additive; FICI > 1.0 and <4, indifferent; FICI > 4.0, antagonism. Synergistic interactions are indicated by gray highlighted cells with bold numbers. Antagonism (FICI > 4) is indicated by orange highlighted cells with bold numbers.

**TABLE 5:** MIC and FICI ranges of double antifungal combinations that showed synergistic interactions against *C. bantiana*, *F. monophora*, and *C. cladosporioides*

Drugs	Characteristics	
	MIC range (µg/mL)	FICI range^*a*^
AmB + 5-FC		0.5–3
AmB	0.25–1	
5-FC	0.06–16	
AmB + VRC		0.38–1.5
AmB	0.25–1	
VRC	0.015–1	
AmB + PSC		0.56–1.5
AmB	0.25–0.5	
PSC	0.015–0.03	
AmB + ISA		0.5–1.5
AmB	0.25–1	
ISA	0.015–0.5	
AmB + CFG		0.5–1.25
AmB	0.25–1	
CFG	0.008–8	
PSC + 5-FC		0.52–3
PSC	0.015–0.25	
5-FC	0.06–1	
ISA + CFG		0.5–2.125
ISA	0.25–1	
CFG	0.008–2	
VRC + MFG		0.5–1.25
VRC	0.25–2	
MFG	0.008–2	
VRC + CFG		0.5–1.25
VRC	0.25–2	
CFG	0.008–1	
ISA + 5-FC		0.5–5
ISA	0.25–2	
5-FC	0.25–16	
ITR + AFG		0.5–1.25
ITR	0.5–1	
AFG	0.015–1	

^*a*^ FICI ≤ 0.5, synergy; FICI > 0.5 and <1.0, partial synergy; FICI = 1.0, additive; FICI > 1.0 and <4, indifferent; FICI > 4.0, antagonism.

**Fig 3:**
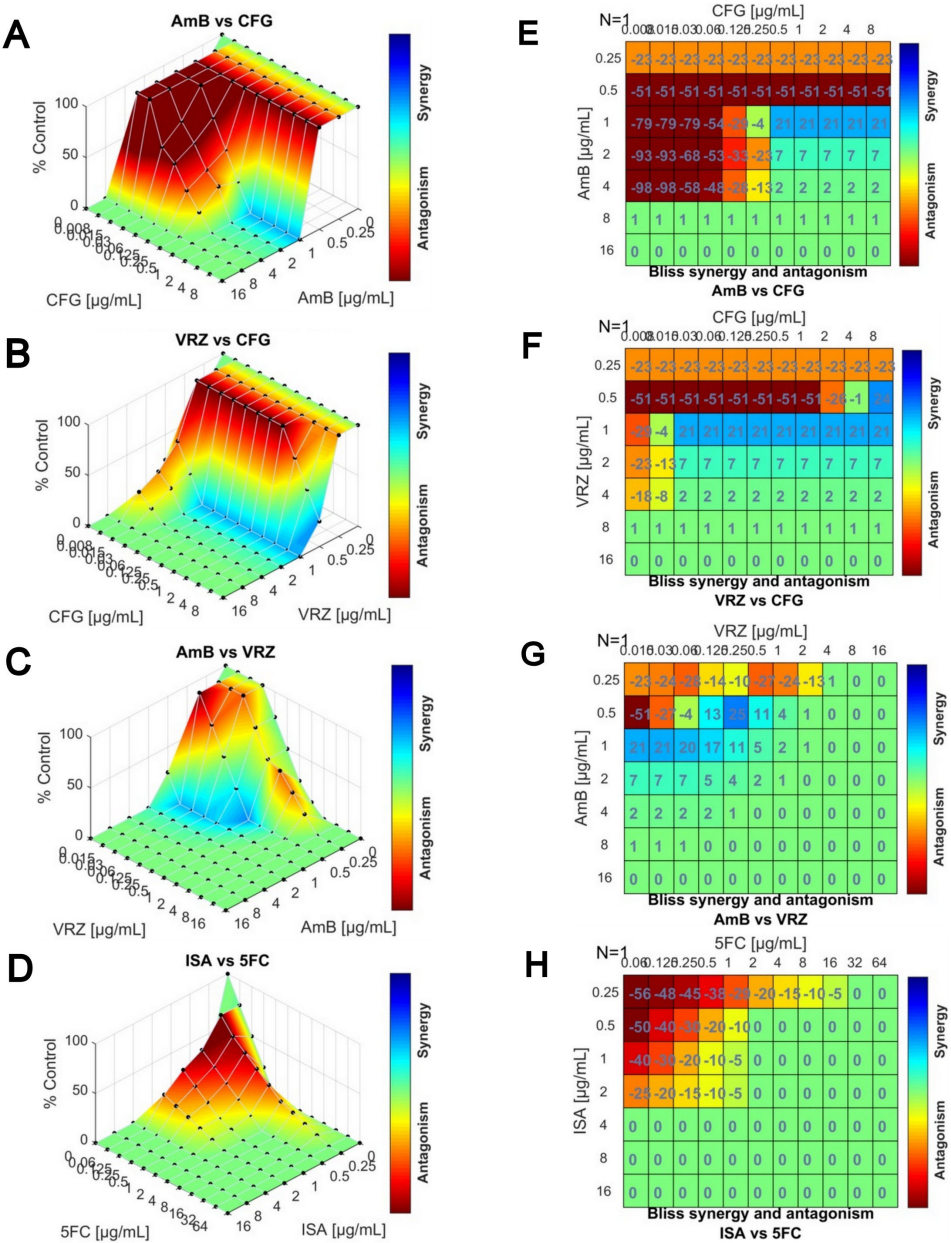
Bliss independence model showing interactions of AmB with CFG or VRZ, VRZ with CFG, and ISA with 5-FC against *C. bantiana* isolate CPPAIIMS. Synergy distributions mapped to dose-response surfaces (**A–D**) and matrix synergy plots (**E–H**) were analyzed with Combenefit v.2.021. The synergy maps depict the growth percentage, relative to the drug-free control, with the color scale representing the drug interaction. A positive score in the matrix plot indicates synergy (blue color), a score of 0 is additive, and a negative score indicates antagonism (red color). Synergistic interactions were observed for the combinations of AmB with CFG or VRZ and VRZ with CFG, while antagonism was noted for the ISA-5-FC combination.

Response surface analyses and matrix synergy plots based on the Bliss Independence model were used to examine the drug interactions, and an example is shown in Fig. 3 for the isolate CPPAIIMS, which demonstrated high individual MICs for AmB and VRC, and high FICIs for 31% of the tested combinations in two-dimensional checkerboard assay. Consistent with the FICI scores, the synergy maps indicated synergy for the combinations of VRC with AmB (FICI, 0.38), CFG (FICI, 0.5), or MFG (0.5), partial synergy for AmB with CFG (FICI, 0.56) and antagonism for ISA with 5-FC (FICI, 5).

### *In vitro* activities of triple antifungal combinations against *C. bantiana*, *F. monophora*, and *C. cladosporioides*

Based on the high individual MICs of AmB, VRC, and echinocandins and the high FICIs observed for 4/13 (31%) double antifungal combinations in two-dimensional checkerboard assay, with ISA-5-FC combination showing antagonism, the isolate *C. bantiana* CPPAIIMS was selected for triple antifungal combination testing against the following combinations: AmB with CFG and 5-FC, VRC with CFG and 5-FC, PSC with CFG and 5-FC, AmB with ITR and 5-FC, and AmB with ITR and AFG. The same combinations were also tested against *F. monophora* (*n* = 2), and *C. cladosporioides* (*n* = 1). The triple combinations showed additive interactions against *C. bantiana* CPPAIIMS and *F. monophora* CPRNIMHANS-02. However, the combinations of CFG and 5-FC with either AmB or PSC displayed synergy against *F. monophora* CPRSRMC-02 (FICI, 0.75). For *C. cladosporioides*, synergy was observed for the triple combination of AmB with CFG and 5-FC (FICI, 0.59), while additive interactions were noted for the other combinations (Table 6).

**TABLE 6:** FICI of five triple antifungal combinations against *C. bantiana*, *F. monophora*, and *C. cladosporioides^a^*

Isolate	MIC (µg/mL) of the drug alone							MIC of drugs in combination				
	AmB	PSC	VRC	ITR	5-FC	CFG	AFG	AmB + CFG + 5-FC	VRC + CFG + 5-FC	PSC +CFG + 5-FC	AmB + ITR + 5-FC	AmB + ITR + AFG
CPPAIIMS	0.5	0.015	0.5	0.5	0.5	8	8	0.25/0.015/2	0.25/0.008/2	0.015/0.008/2	0.25/0.5/2	0.25/0.5/0.015
FICI								1.502	1.5	2	2.5	1.5
CPRSRMC-02	2	0.06	0.25	0.5	16	8	8	0.5/0.008/8	0.25/0.25/8	0.015/0.008/8	0.5/0.5/8	0.5/0.25/0.25
FICI								**0.75**	1.53	**0.75**	1.75	0.78
CPRNIMHANS-02	1	0.015	0.25	1	4	8	8	1.0/0.5/2	0.25/0.5/2	0.015/0.125/2	1.0/0.5/2	1.0/0.5/0.5
FICI								1.56	1.56	1.52	2	2.06
CPRNIMHANS-01	1	0.06	0.5	1	64	8	8	0.25/0.5/2	0.5/0.5/2	0.06/0.25/2	0.5/1/2	0.25/0.5/1
FICI								**0.59**	1.09	1.06	1.53	0.875

^*a*^ FICI ≤ 0.75, synergy; FICI > 0.75–3, additive; FICI > 3–4, indifferent; and FICI > 4, antagonism. Synergistic interactions are indicated by gray highlighted cells with bold numbers.

## DISCUSSION

In this study, we evaluated the *in vitro* activities of nine major antifungal agents and their combinations against *C. bantiana*, *F. monophora*, and *C. cladosporioides*, three neurotropic dematiaceous fungi commonly associated with CPHM in India. Our results showed that PSC (MIC range: 0.015–0.06 µg/mL) has the most potent and consistent activity against this group of fungi. PSC is an extended-spectrum triazole antifungal with good *in vitro* activity against *Mucorales*, *Aspergillus* spp., *Cryptococcus* spp., *Scedosporium apiospermum*, *Coccidioides immitis*, *Histoplasma capsulatum*, and *Blastomyces dermatitidis* (11). The drug has been found to reduce the brain fungal burden and improve survival in experimental animal models of CPHM, involving *C. bantiana* (12), *R. mackenziei* (13), *E. dermatitidis* (14), and *F. monophora* (15). It has also been found to be effective in refractory cases of CPHM and has supplanted AmB for this indication (16). Among other triazoles, ITR demonstrated potent activity (MIC range: 0.5–1 µg/mL) against all the tested isolates, which is consistent with the results of previous studies (17, 18). While VRC achieves excellent penetration into the CSF and brain parenchyma and displays good activity against most agents of CPHM (19, 20), variable activity was noted against some of our isolates, indicating that VRC might not be the drug of choice for the treatment of such infections. Also, there are reports of clinical failure with VRC in several cases of *C. bantiana* brain abscesses (20, 21). These observations underscore the importance of *in vitro* susceptibility testing before using VRC for the treatment of CPHM. ISA is a broad-spectrum triazole that was approved by the FDA for the treatment of invasive aspergillosis and mucormycosis. The drug has a pharmacokinetic profile similar to that of fluconazole and has fewer drug interactions than VRC and ITR (22). Although antifungal activities of ISA have been well studied *in vitro* and animal models of invasive aspergillosis, mucormycosis, candidiasis, endemic mycoses, and other invasive fungal diseases (23), *in vitro* susceptibility data for dematiaceous fungi are relatively limited, and its clinical efficacy in the treatment of CPHM has not been evaluated. In the present study, ISA exhibited variable susceptibility against *C. bantiana*; however, good activity was observed against *F. monophora* and *C. cladosporioides*. This agrees with the *in vitro* data from previous studies that demonstrated ISA as a potential treatment option for infections caused by *Fonsecaea* spp. (24), and *Cladosporium* spp. (25). Fluconazole has negligible activity against dematiaceous fungi and essentially no role in therapy (1, 4, 12, 26). Therefore, fluconazole was not used for susceptibility testing in this study. The triazoles demonstrated the broadest overall activity against agents of CPHM. Although none of the azoles have been independently associated with improved outcomes, several other factors, such as time to diagnosis, site of CNS lesion, scope of surgical excision, and *in vitro* susceptibility to the antifungal drug are important predictors of survival (4). Prospective studies are required to evaluate the efficacy of high-dose triazole-based regimens in the treatment of CPHM.

AmB is the cornerstone in the management of CNS fungal infections and has been the most commonly (63%) used antifungal agent for the treatment of CPHM (3, 4). Although the drug has excellent penetration into the brain and displays good *in vitro* activity against most clinically relevant dematiaceous fungi, it was ineffective in several cases of CPHM (21, 27). This was evident even in experimental murine models of CPHM due to *C. bantiana*, where triazoles were found to be more effective than AmB (12, 28). *In vitro* results indicate that AmB MICs for most opportunistic filamentous fungi range between 0.5 and 2 µg/mL (27). However, there is limited data on the correlation between MIC and treatment outcomes with AmB for dematiaceous fungi. In our study, high MIC of AmB was observed for one isolate each of *C. bantiana*, and *F. monophora*, indicating variable *in vitro* susceptibility of this group of fungi to this antifungal agent. Similar findings were reported by others (14, 17).

5-FC is a nucleotide analog with a broad spectrum of antifungal activity and excellent penetration into the CSF. Several *in vitro* studies and animal models have shown good activity of 5-FC against dematiaceous fungi, in particular *C. bantiana* (12, 26), as noted in our isolates. However, high MIC values were observed for *F. monophora* (MIC range: 4–16 µg/mL), and *C. cladosporioides* (64 µg/mL), which were consistent with other studies (29, 30), indicating that the drug is ineffective against these species, discouraging its use as monotherapy in such cases.

Echinocandins have a limited role in the treatment of CPHM due to poor penetration into the CSF and high MEC values shown by most species of dematiaceous fungi (4, 24). They exhibit a variable and species-dependent fungistatic activity against this group of fungi. Our results showed consistently high MECs of CFG and AFG against all the tested isolates. However, better *in vitro* responses were observed for MFG against *C. bantiana* and *F. monophora*, which is in accordance with previous studies (1, 31). Overall, the therapeutic success of echinocandins is meager and their use in CPHM is recommended only in combination with azole and flucytosine when surgery is not possible (5).

Combination therapies provide a reasonable strategy for preventing the emergence of drug resistance and have already been employed in the treatment of bacterial (32) and viral infections (33). They have the potential to improve efficacy through synergistic or additive interactions, allowing for a lowering of doses, and thereby reducing dose-related toxicity. Antifungal combinations are being increasingly evaluated for the treatment of refractory fungal infections (34, 35). The checkerboard assay is a well-established tool for determining the interactions between two or more drugs in combination. The existing checkerboard methods for testing antifungal combinations are cumbersome, labor-intensive, and time-consuming, which limit their application in routine mycology laboratories (9). In this study, we developed a simplified and cost-effective checkerboard method for testing antifungal combinations, which can easily be adopted by laboratories without the need for special equipment. Currently, there is no optimal antifungal combination for the treatment of CPHM. Though several regimens have been described in the literature (5, 35, 36), the *in vitro* effectiveness of antifungal combinations against neurotropic dematiaceous fungi has seldom been investigated. Nearly all successfully treated cases of CPHM received a combination of antifungal agents (2–4). Monotherapy might seem effective initially but is almost always associated with treatment failure (2). The combination of AmB with 5-FC has been traditionally used for the treatment of CNS fungal infections, particularly cryptococcal meningitis, due to the favorable pharmacokinetic profile of flucytosine (7). There is limited data on the *in vitro* efficacy of this combination against agents of CPHM. Studies have shown consistent synergistic interactions of AmB-5FC combination against *C. bantiana, R. mackenziei, E. dermatitides*, and *F. monophora* (29, 37). However, the combination failed to demonstrate any favorable activity against our isolates, except for *C. cladosporioides*. We found synergistic interactions when AmB was combined with either CFG or azoles (VRC, PSC, and ISA). Importantly, the combination of AmB and VRC displayed synergy against an isolate of *C. bantiana* (CPPAIIMS), which exhibited high MIC for either drug alone. According to the FICI values and response-surface analyses, the most potent combination (in terms of the number of isolates that showed synergy) was AmB plus CFG, and this was followed by the combinations of VRC with CFG, and ITR with AFG. In contrast to FICI, which measures drug interactions based only on the corresponding MIC values, the response-surface analysis and matrix synergy plot permit evaluation of drug interactions over a wide range of tested concentrations. Thus, antagonism was demonstrated at the lower end of some concentration ranges that were otherwise missed by the FICI approach, highlighting the concentration-dependent nature of the interactions.

Echinocandins have been shown to interact synergistically with azoles *in vitro* against diverse fungal pathogens, including *Candida albicans*, *Aspergillus* spp., *Fonsecaea* spp., *Phialophora verrucosa*, and *Sporothrix schenckii* (38–40). The type of interaction exhibited by an echinocandin-azole combination depends on the azole tested (41). In our study, synergistic interactions were observed for all the echinocandin-azole combinations, with VRC-CFG and ITR-AFG showing synergy against a maximum number of isolates. Our findings are consistent with other studies (5, 36), indicating that the combination of an echinocandin with a triazole could be a promising treatment option for CPHM.

The combination of 5-FC with azoles, particularly fluconazole, has been extensively evaluated in experimental models (42–44), and clinical trials of cryptococcal meningitis (45, 46). Based on its *in vitro* synergy and *in vivo* efficacy against *C. neoformans*, the 5-FC-fluconazole combination, along with single high-dose liposomal AmB is currently recommended as the first-line therapy for cryptococcal meningitis (47). However, the 5-FC-azole synergy is not universal and there are reports of antagonistic interactions between 5-FC and azoles against specific fungal pathogens (48, 49). It was observed that the combination of 5-FC with fluconazole was antagonistic against *C. glabrata*, possibly due to Pdr1 (positive regulator of proteins involved in permeability) activation mediated by mitochondrial dysfunction (49). In another study, the 5-FC-fluconazole combination demonstrated antagonism against *C. lusitaniae* due to competitive inhibition of the fluconazole uptake transporter (50). In our study, the combination of 5-FC with ISA resulted in antagonistic interactions against two isolates of *C. bantiana*. However, the possible mechanism underlying such antagonism remains to be investigated.

Combinations of more than two antimicrobial agents are commonly used for difficult-to-treat infections, such as tuberculosis (32), and human immunodeficiency virus infection (33). These conditions require long-term treatment with antimicrobials and are prone to relapse or develop drug resistance. Therefore, combination regimens consisting of different classes of antimicrobials, having different mechanisms of action are used to improve the efficacy and prevent the emergence of resistance to a single agent (51). In medical mycology, triple antifungal combinations have not been evaluated properly either *in vitro* or in experimental animals and have rarely been used in clinical practice. Mariné et al. studied the *in vivo* efficacy of triple antifungal combinations in an experimental murine model of disseminated *C. bantiana* infection and observed that the PSC-MFG-5FC combination was associated with a significant reduction in brain fungal burden and improved survival (28). In clinical practice, the use of triple antifungal therapy has been largely confined to cryptococcal infections (46), and inoperable cases of *C. bantiana* brain abscess (5). To date, the triple antifungal combinations have been evaluated *in vitro* only against *Candida* spp. (52, 53), and *Aspergillus* spp. (35, 54, 55), while the effectiveness of these combinations against neurotropic dematiaceous fungi has not been investigated. In the present study, synergistic interactions were observed with the triple combinations of AmB-CFG-5FC and PSC-CFG-5FC against *F. monophora*, with the former showing synergy against *C. cladosporioides*, as well. The double combinations of these drugs demonstrated additive to synergistic interactions with no evidence of antagonism, and this could be a possible explanation for the synergy observed when the three drugs were tested in combination. The other combinations showed additive interactions against the tested isolates.

One of the limitations of this study was the relatively small number of isolates tested. Moreover, the results of *in vitro* drug combination assays may not correlate with *in vivo* efficacy against this group of fungi. The antifungal susceptibility patterns are highly variable among studies and a definite interpretive breakpoint is difficult to establish. This is related in part to the relative rarity of the disease and the lack of a standardized method for studying the *in vitro* activities of drug combinations. Robust preclinical studies are required to generate sufficient data on antifungal combination regimens before clinical trials with humans can be designed. Our results underscore the need for animal experiments to explicate the clinical implications of these complex interactions observed *in vitro*.

### Conclusions

We developed a simplified checkerboard method for testing antifungal combinations that can easily be adopted by routine mycology laboratories for *in vitro* drug combination studies. Our results indicate that PSC has the most potent *in vitro* activity against neurotropic dematiaceous fungi and can be used as the first-line therapy for CPHM. A combination of drugs shows more potent inhibitory activity than a single agent. Among the double combinations, AmB with CFG has the maximum synergistic activity against neurotropic dematiaceous fungi. The combination of a trizole with either AmB or an echinocandin can be a reasonable alternative in such cases. In contrast, the combination of ISA and 5-FC can be antagonistic and should be avoided. The triple antifungal combinations consisting of an echinocandin, 5-FC, and either AmB or a triazole have better *in vitro* activity against this group of fungi than double combinations. The findings of our study might help in developing optimal therapeutic strategies for these refractory infections.

## Data Availability

The raw sequence data reported in this study have been deposited in the GenBank database of the National Center for Biotechnology Information under the accession numbers from PP462145 to PP462154.
